# Deubiquitinase PSMD7 promotes the proliferation, invasion, and cisplatin resistance of gastric cancer cells by stabilizing RAD23B

**DOI:** 10.7150/ijbs.61128

**Published:** 2021-07-25

**Authors:** Jianjiang Wang, Runkun Liu, Huanye Mo, Xuelian Xiao, Qiuran Xu, Wei Zhao

**Affiliations:** 1 Department of Hepatobiliary Surgery, The First People's Hospital of Hangzhou Lin'an District, Affiliated Lin'an People's Hospital, Hangzhou Medical College, Hangzhou 311399, China; 2 Department of Hepatobiliary Surgery, The First Affiliated Hospital of Xi'an Jiaotong University, Xi'an 710061, China; 3 The Key Laboratory of Tumor Molecular Diagnosis and Individualized Medicine of Zhejiang Province, Zhejiang Provincial People's Hospital, Affiliated People's Hospital, Hangzhou Medical College, Hangzhou 310014, China; 4 Department of General Surgery, The First Affiliated Hospital of Xi'an Jiaotong University, Xi'an 710061, China

**Keywords:** Gastric cancer, PSMD7, RAD23B, DUBs, Chemoresistance

## Abstract

Ubiquitination, a crucial post-translational modification, controls substrate degradation and can be reversed by deubiquitinases (DUBs). An increasing number of studies are showing that DUBs regulate the malignant behavior and chemotherapy resistance of gastric cancer (GC) by stabilizing various proteins. However, the expression level and biological function of the DUB, proteasome 26S subunit, non-ATPase 7 (PSMD7), in GC remains unknown. Herein, we report for the first time that PSMD7 is frequently overexpressed in GC tissues. Elevated levels of PSMD7 were also detected in GC cell lines. Notably, the upregulation of PSMD7 closely correlated with malignant clinical parameters and reduced the survival of GC patients. Functionally, we found that PSMD7 knockdown consistently suppressed the proliferation, migration, and invasion of AGS and SGC-7901 cells. Ectopic expression of PSMD7 facilitated GC cell proliferation and mobility. Based on protein-protein interaction prediction, RAD23 homolog B (RAD23B) protein was identified as a candidate substrate of PSMD7. PSMD7 positively regulated the abundance of RAD23B and xeroderma pigmentosum, complementation group C (XPC) protein in GC cells. The interaction between PSMD7 and RAD23B was confirmed using protein immunoprecipitation. PSMD7 knockdown enhanced the ubiquitination and degradation of RAD23B protein in GC cells. PSMD7 promoted cell viability, apoptosis resistance, and DNA damage repair in GC cells upon cisplatin (DDP) treatment. Moreover, PSMD7 silencing inhibited tumor growth and enhanced the sensitivity of GC cells to DDP treatment in mice. In summary, PSMD7 was highly expressed in GC and contributed to the malignant behavior and DDP resistance of tumor cells by stabilizing RAD23B.

## Introduction

Recently, the International Agency for Research on Cancer (IARC) released updated data for the 2020 global cancer burden, indicating that gastric cancer (GC) has become the fifth most common cancer and the fourth leading cause of cancer-related deaths worldwide^1^. In China, the incidence and mortality of GC ranks second among those of all malignancies^2^. Surgical resection is the best choice for the treatment of GC patients with localized disease. Chemotherapy has improved the clinical outcomes of patients with advanced GC. However, the treatment effects are limited due to drug resistance. Therefore, it is essential to determine the pathogenesis of GC to develop new therapeutic targets and explore novel biomarkers for predicting poor prognosis.

Protein stability is mainly controlled by ubiquitination and deubiquitination^3^-^5^. Ubiquitinating enzymes, including E1, E2, and E3 ligases, are required for the attachment of Ub to a substrate^6^, ^7^. Deubiquitinases (DUBs) are responsible for removing Ub chains from the substrate and enhancing protein stability^8^. To date, no less than 100 genes that encode DUBs have been identified in the human genome. Several studies have confirmed that DUBs play essential roles in the occurrence and progression of human cancers, and have thus provided emerging drug discovery opportunities^9^-^12^. In GC, several DUBs participate in tumor initiation and progression. For instance, a high level of ubiquitin-specific protease 29 (USP29) indicates poor prognosis of GC and contributes to cancer cell migration by stabilizing Snail protein^13^. USP3, which is induced by transforming growth factor-beta 1 (TGF-β1), facilitates the migration and invasion of GC cells by enhancing the deubiquitination and stability of suppressor of Zeste 12 homolog (SUZ12)^14^. Moreover, USP7 deubiquitinates and stabilizes heterogeneous nuclear ribonucleoprotein A1 (hnRNPA1) to enhance the secretion of cancer-associated fibroblast (CAF)-derived exosomes, which transfer miR-522 to enhance the chemoresistance of GC cells^15^. Proteasome 26S subunit, non-ATPase 7 (PSMD7), an ATP-independent component of the 19S regulatory subunit, is a member of the JAMM/MPN domain-associated metallopeptidase (JAMM) DUB family^9^, ^16^. Recent studies have revealed the role of PSMD7 in prostate cancer, esophageal squamous cell carcinoma (ESCC), and breast cancer^17^-^19^. PSMD7 rs2387084 is prominently associated with earlier onset of prostate cancer and advanced clinical stages^17^. *PSMD7* was identified as an oncogene in ESCC, and knockdown of PSMD7 induced proliferation inhibition, apoptosis, and mechanistic target of rapamycin (mTOR)/p70S6K pathway inactivation^18^. PSMD7 expression is upregulated in breast cancer and acts as an independent prognostic factor for predicting the overall and disease-free survival of patients^19^, ^20^. PSMD7 promotes cell cycle progression and suppresses cell senescence and apoptosis of breast cancer cells by regulating the stability of p21 and p27^19^. However, the clinical significance and biological function of PSMD7 in GC remain unknown.

In this study, we determined the difference in PSMD7 expression in GC and adjacent non-tumor tissues. The clinical significance and prognostic value of PSMD7 in GC were analyzed. We assessed the regulatory effects of PSMD7 on the proliferation, migration, invasion, and cisplatin (DDP) resistance of GC cells. Finally, the interaction between PSMD7 and RAD23 homolog B (RAD23B) and the associated regulatory mechanism were investigated. Our data suggest that *PSMD7* is a novel oncogene in GC, and it contributes to the malignant behavior and DDP resistance of cancer cells by deubiquitinating and stabilizing RAD23B.

## Material and methods

### Clinical samples

GC and tumor-adjacent tissues were harvested from patients who underwent R0 resection at the 1^st^ Affiliated Hospital of Xi'an Jiaotong University after obtaining written informed consent. None of the patients received any treatment before surgery and all had complete medical records and follow-up data. All the specimens were fixed with 10% formalin and subjected to pathological examination or preserved at -80 °C. The Ethics Committee of the 1^st^ Affiliated Hospital of Xi'an Jiaotong University approved the protocols of this study. Table 1 lists the clinical parameters of the GC patients.

### Cell culture, transfection, and treatment

The human gastric cancer cell lines, AGS, SGC-7901, MGC-803, and MKN-45, and the gastric mucosal cell line, GES-1, were maintained and cultured under standard conditions in our lab^21^. The expression vectors, PSMD7-OE and RAD23B-OE, were constructed by inserting the cDNA products of PSMD7 and RAD23B into the pcDNA3.1(+) vector (V79020, Invitrogen, Carlsbad, CA, USA) and transfected into GC cells using Effectene transfection reagent (301427, Qiagen, Valencia, CA, USA). For PSMD7 shRNAs (shPSMD7-1 and shPSMD7-2) and non-targeting (NT) shRNA, pLKO.1-PSMD7/NT shRNA (2.0 μg), pVSV.G (0.5 μg), and p8.91 (1.5 μg) were co-transfected into 293T cells using Effectene transfection reagent. Lentiviruses were harvested at 48 and 72 h post-transfection and used to infect GC cells with polybrene (8 μg/mL; 40804ES76, YEASEN, Shanghai, China). The GC cells were then treated with cycloheximide (CHX; 66-81-9, Sigma-Aldrich, St. Louis, MO, USA), carbobenzoxy-Leu-Leu-leucinal (MG132; 1211877-36-9, Sigma-Aldrich), or DDP (HY-17394, MedChem Express, Monmouth Junction, NJ, USA) for different experiments.

### Quantitative real-time polymerase chain reaction (qRT-PCR)

RNA extraction, reverse transcription, and PCR amplification were performed using TRIzol reagent (15596018, Invitrogen), the TIANScript RT Kit (KR104, Tiangen Biotech, Beijing, China), FS Universal SYBR Green Master (4913850001, Roche, Shanghai, China), and the CFX96 Touch™ real-time PCR detection system (Bio-Rad Laboratories, Hercules, CA, USA), following the manufacturer's instructions. The expression of PSMD7 and RAD23B mRNA relative to β-actin was calculated using the 2^-ΔΔCt^ method. Supplementary Table 1 lists the primer sequences used in this study.

### Western blot (WB)

Protein isolation and concentration detection were carried out using radioimmunoprecipitation assay (RIPA) lysis buffer (P0013B, Beyotime, Shanghai, China) containing protease and phosphatase inhibitors (P1056, Beyotime) and the Bradford protein assay kit (P0006, Beyotime), respectively. Then, 10% sodium dodecyl sulfate-polyacrylamide gel electrophoresis (SDS-PAGE) was used to separate the proteins, which were transferred to a polyvinylidene fluoride (PVDF) membrane (IPVH00010, Millipore, Bedford, MA, USA). The membranes were incubated overnight at 4 °C with anti-PSMD7 (1:1500, 16034-1-AP, Proteintech, Wuhan, China), anti-RAD23B (1:1000, 12121-1-AP, Proteintech), anti-xeroderma pigmentosum, complementation group C (XPC; 1:1000, #14768, Cell Signaling Technology, Beverly, MA, USA), anti-ubiquitin (1:1000, PTM-1105, PTM BIO, Hangzhou, China), anti-phosphorylated histone H2AX (γ-H2AX; 1:1000, ab26350, Abcam, Cambridge, MA, USA), and anti-β-actin (1:5000, 66009-1-Ig, Proteintech). Next, the non-specifically bound primary antibody on the membrane was washed, and the horseradish peroxidase (HRP)-bound secondary antibodies (1:1000, A0208 and A0216, Beyotime) were added to the membrane and incubated. Immunoreactive proteins were detected using an electrochemical luminescence (ECL) system (WBLUF0500, Millipore). The resulting bands were scanned using an Amersham Imager 680 (GE Healthcare Life Sciences, Pittsburgh, PA, USA).

### Immunohistochemistry (IHC) analysis

Formalin-fixed and paraffin-embedded GC and tumor-adjacent tissues were used. IHC was performed on 4-μm-thick routinely processed paraffin sections. After deparaffinization and rehydration, the tissue sections were heated with sodium citrate buffer for antigen restoration. The sections were then incubated with anti-PSMD7 (ab11436, Abcam), anti-RAD23B (12121-1-AP, Proteintech), anti-XPC (ab155025, Abcam), and anti-Ki-67 (27309-1-AP, Proteintech) at 4 °C for 24 h. Finally, 3,3-diaminobenzidine (DAB) was used for staining. The percentage of stained cells (1: <25%, 2: 25%-75%, 3: >75%) was multiplied by the staining intensity (0, 1, 2, or 3) to calculate the final IHC score (0-9), for which 3 or higher was considered to indicate positive PSMD7 expression.

### Cell proliferation assay

The proliferation of GC cells was assessed using the thiazolyl blue tetrazolium bromide (MTT) assay with the MTT reagent (CT02, Sigma-Aldrich) and the 5-ethynyl-2′-deoxyuridine (EdU) assay with the Cell-Light™ EdU Apollo®488 *In vitro* Imaging Kit (C10310-1, RiboBio, Guangzhou, China), as previously described^21^.

### Transwell migration and invasion assays

The migration and invasion potentials of GC cells were measured using Transwell chambers (BD Biosciences, Franklin Lakes, NJ, USA) with or without Matrigel coating (356234, BD Biosciences). GC cells (3 × 10^4^) suspended in serum-free Dulbecco's Modified Eagle Medium (DMEM) were added to the upper chamber. Next, DMEM containing 20% fetal bovine serum (FBS) was added as a chemoattractant to the lower chamber. The cells were then incubated for 48 h. Crystal violet was used to stain the cells that penetrated or invaded the membrane. Images of the membrane area were captured randomly, and the cells were counted.

### Apoptosis assay

According to the manufacturer's protocols, the percentage of apoptotic GC cells was detected using the PE Annexin V Apoptosis Detection Kit I (#559763, BD Biosciences).

### Co-immunoprecipitation (co-IP) assay

GC cells were lysed with an IP buffer containing 0.5% NP40. GC cell lysates containing equal volumes and equal amounts of proteins were incubated with 3 µg anti-PSMD7 (16034-1-AP, Proteintech) and 30 µL slurry of Protein G Sepharose (GE17-0618-01, GE Healthcare) overnight to pull down PSMD7 and its associated proteins. The beads were precipitated by centrifugation on the following day, and RAD23B in the precipitates was quantitated by WB using anti-RAD23B (sc-390019, Santa Cruz Biotechnology, Santa Cruz, Dallas, TX, USA). For ubiquitination detection, RAD23B was pulled down using 2 µg anti-RAD23B (1:1000, sc-390019, Santa Cruz Biotechnology), and ubiquitinated RAD23B was determined by WB using anti-ubiquitin (1:1000, PTM-1105, PTM BIO).

### Mouse xenograft model

All animal procedures were performed according to a protocol approved by the Institutional Animal Care and Use Committee of Xi'an Jiaotong University (Approval number: 2019-058). An experimental mouse xenograft model was established by subcutaneous injection of 5 × 10^6^ GC cells with or without PSMD7 knockdown (*n* = 5 per group) in 100 μL of PBS into 6-week-old female nude mice. Tumor volumes were recorded every 4 days and calculated according to the following formula: volume = (width^2^ × length)/2. One week after implantation, the mice were treated with DDP (5 mg/kg) in PBS (thrice a week) via intraperitoneal injection. Four weeks after implantation, the mice were sacrificed, and xenograft tumors were collected for IHC and terminal deoxynucleotidyl transferase dUTP nick end labeling (TUNEL) assay (ab206386, Abcam) following the manufacturer's instructions.

### Statistical analysis

Data were expressed as means ± standard deviations (SD), and each experiment was repeated at least thrice. GraphPad Prism version 8 (GraphPad Inc., San Diego, CA, USA) was used to statistically analyze the results. The Mann-Whitney test and one-way analysis of variance (ANOVA) with Tukey's multiple comparison test were used to assess the significance of differences between groups. The chi-square test and Fisher's exact test were used appropriately to calculate the relationship between PSMD7 expression and the clinical characteristics of GC. The Kaplan-Meier method was used to calculate the survival rate, and the log-rank test was used to compare survival curves. Statistical significance was set at *P* < 0.05.

## Results

### PSMD7 is highly expressed in GC tissues and is associated with a poor prognosis

**Figure 1:**
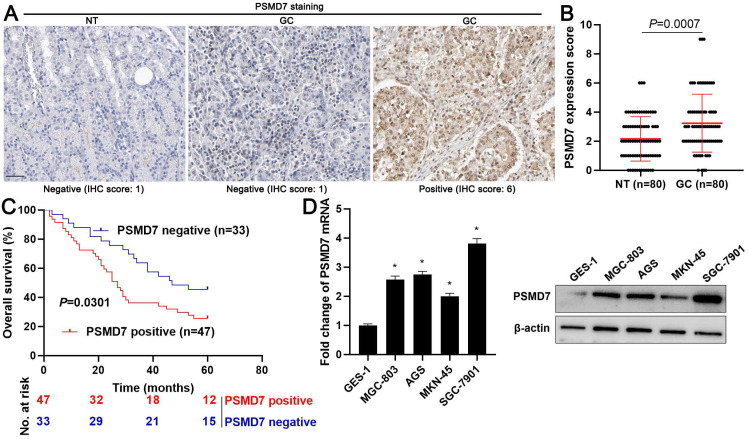
** Expression and prognostic significance of PSMD7 in GC.** (A) Representative IHC staining image of PSMD7 in GC and tumor-adjacent tissues. Scale bar: 50 μm. (B) IHC scores of PSMD7 in GC and adjacent non-tumor tissues. (C) Survival analysis of GC patients with positive or negative PSMD7 expression. (D) Levels of PSMD7 in the GC cell lines, MGC-803, AGS, MKN-45, and SGC-7901, and the gastric mucosal cell line, GES-1. *P < 0.05.

Firstly, we screened the expression and prognostic value of 105 DUBs in GC from The Cancer Genome Atlas (TCGA) data using the Gene Expression Profiling Interactive Analysis (GEPIA) web server^22^. We found that PSMD7 mRNA expression was frequently upregulated in GC tissues and correlated with poor prognosis (*P* < 0.05, Supplementary Figure 1). PSMD7 protein levels were detected in 80 pairs of GC and tumor-adjacent tissues using IHC staining. We found that 47 of 80 (58.75%) GC tissues showed positive PSMD7 expression, whereas only 40.00% of adjacent non-tumor tissues positively expressed PSMD7 (*P* = 0.018, Figure 1A). The quantitative analysis of PSMD7 IHC scores further supported the overexpression of PSMD7 in GC tissues (*P* = 0.0007, Figure 1B). As shown in Table 1, the positive expression of PSMD7 was associated with poor differentiation (*P* = 0.019), tumor size ≥ 5 cm (*P* = 0.011), and the advanced tumor-node-metastasis (TNM) stage (*P* = 0.043). Importantly, our data revealed that GC patients with positive PSMD7 expression exhibited a markedly lower overall survival compared with those with negative PSMD7 expression (*P* = 0.0301, Figure 1C). Moreover, the levels of PSMD7 in the GC cell lines, MGC-803, AGS, MKN-45, and SGC-7901, were consistently higher than those in the gastric mucosal cell line, GES-1 (*P* < 0.05, Figure 1D). These data indicated that *PSMD7* is a potential oncogene in GC.

### PSMD7 promotes the proliferation, migration, and invasion of GC cells

**Figure 2:**
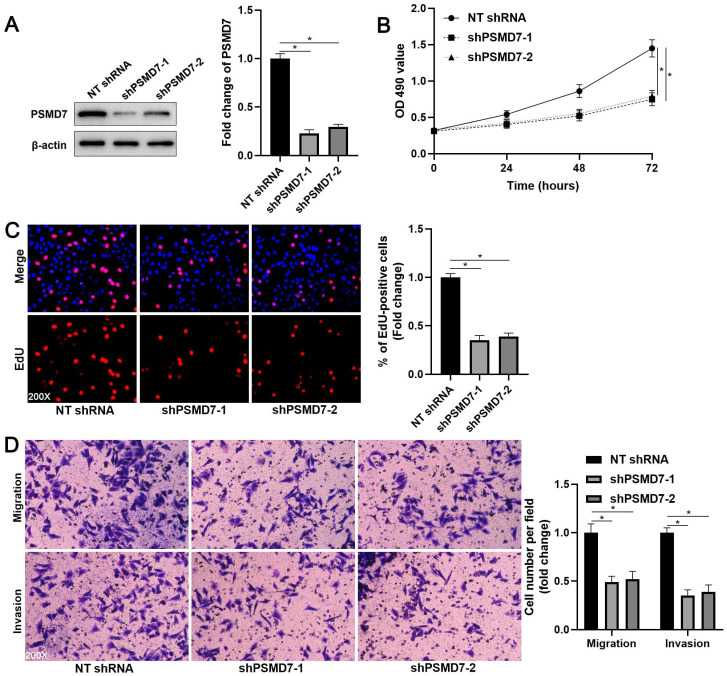
** PSMD7 knockdown inhibits the proliferation, migration, and invasion of AGS cells.** (A) AGS cells were infected with lentiviruses carrying non-targeting (NT) shRNA or PSMD7 shRNA (shPSMD7-1 and shPSMD7-2) and analyzed by WB for PSMD7 expression. (B) MTT assay confirmed that PSMD7 knockdown repressed the viability of AGS cells. (C) EdU assay verified that PSMD7 silencing decreased the proliferation of AGS cells. (D) The migration and invasion potentials of AGS cells were suppressed by PSMD7 knockdown. *P < 0.05.

**Figure 3:**
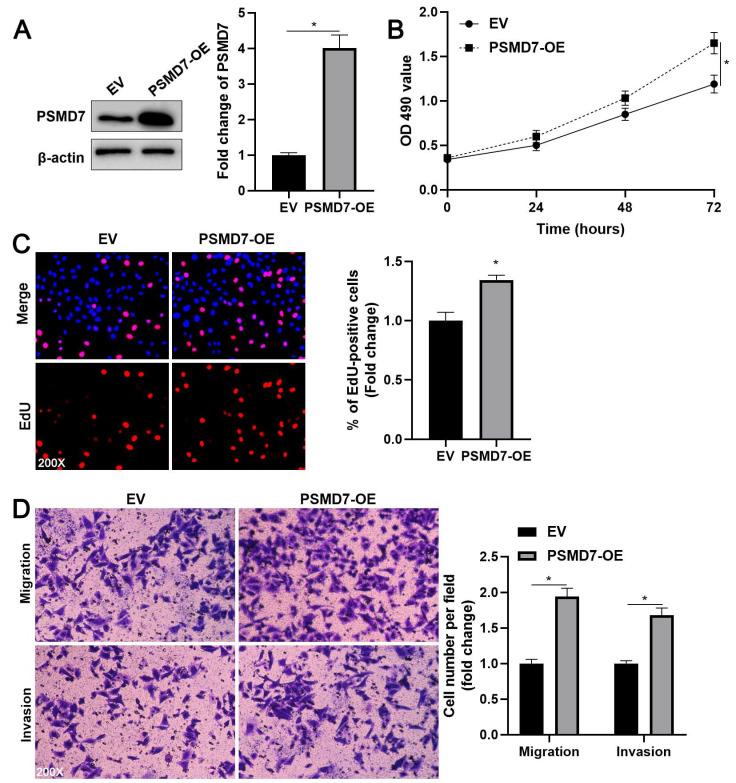
** PSMD7 overexpression facilitates AGS cell proliferation, migration, and invasion.** (A) AGS cells were transfected with pcDNA3.1 vector carrying PSMD7 cDNA or an empty vector and analyzed by WB for PSMD7 expression. (B) MTT analysis revealed that PSMD7 overexpression enhanced the viability of AGS cells. (C) EdU analysis indicated that ectopic expression of PSMD7 increased the proliferation of AGS cells. (D) The migration and invasion potentials of AGS cells were enhanced by PSMD7 overexpression. *P < 0.05.

Next, we verified the regulatory effects of PSMD7 on GC cell proliferation, migration, and invasion. We downregulated PSMD7 expression in AGS cells by transfecting two independent shRNA constructs (*P* < 0.05, Figure 2A). MTT and EdU assays consistently demonstrated that PSMD7 knockdown reduced AGS cell proliferation (*P* < 0.05, Figures 2B and 2C). Furthermore, transwell assays indicated that PSMD7 silencing significantly inhibited AGS cell migration and invasion (*P* < 0.05, Figure 2D). The inhibitory effects of PSMD7 knockdown on cell proliferation, migration, and invasion were further confirmed using SGC-7901 cells (*P* < 0.05, Supplementary Figure 2). In contrast, we upregulated PSMD7 expression in AGS and MKN-45 cells via plasmid transfection (*P* < 0.05, Figure 3A and Supplementary Figure 3A). The ectopic expression of PSMD7 significantly facilitated the proliferation, migration, and invasion of AGS and MKN-45 cells (*P* < 0.05, Figures 3B-3D and Supplementary Figures 3B-3D). Therefore, PSMD7 contributed to the malignant behavior of GC cells.

### PSMD7 enhances the stability of RAD23B via deubiquitination

**Figure 4:**
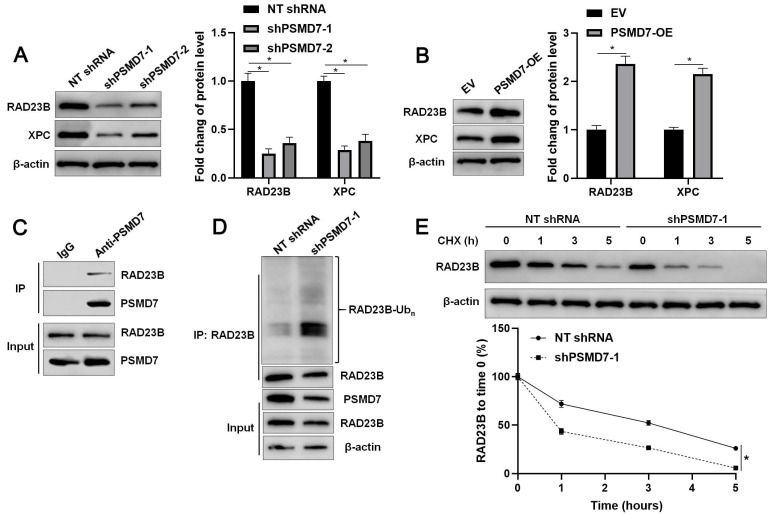
** PSMD7 stabilizes RAD23B via deubiquitination in GC cells.** (A) AGS cells were infected with lentiviruses carrying non-targeting (NT) shRNA or PSMD7 shRNA (shPSMD7-1 and shPSMD7-2) and analyzed by WB for RAD23B and XPC expression. (B) AGS cells were transfected with pcDNA3.1 vector carrying PSMD7 cDNA or an empty vector and analyzed by WB for RAD23B and XPC expression. (C) Co-IP assay confirmed the interaction between PSMD7 and RAD23B in AGS cells. (D) PSMD7 knockdown increased the ubiquitination of RAD23B in AGS cells. (E) AGS cells with or without PSMD7 silencing were treated with CHX (40 mg/mL) to block protein synthesis. WB was carried out to detect the RAD23B level 0, 1, 3, and 5 h after treatment. *P < 0.05.

We aimed to investigate the potential substrate of PSMD7, a DUB, in GC. We firstly predicted the proteins that interacted with PSMD7 using the human protein-protein interaction prediction (PIP) web server^23^, ^24^. Most of the predicted proteins were proteasome regulatory subunits, except for RAD23B, the gene for which was previously identified as an oncogene in GC^25^, ^26^. The interaction between PSMD7 and RAD23B was further confirmed using the STRING database (Supplementary Figure 4)^27^. Then, we found that PSMD7 knockdown reduced, whereas PSMD7 overexpression increased, RAD23B protein levels in GC cells (*P* < 0.05, Figures 4A and 4B, and Supplementary Figures 3A and 5A). However, altering the PSMD7 level did not affect the level of PSMD7 mRNA in AGS and SGC-7901 cells (Supplementary Figure 5B). PSMD7 positively regulated the abundance of XPC protein, a downstream regulatory target of RAD23B, in GC cells (*P* < 0.05, Figures 4A and 4B, and Supplementary Figures 3A and 5A). IHC staining indicated that RAD23B and XPC protein levels in PSMD7-positive GCs were significantly higher than those in PSMD7-negative GCs (*P* < 0.05, Supplementary Figure 6). The co-IP assay demonstrated that PSMD7 interacted with RAD23B in GC cells (Figure 4C). PSMD7 knockdown increased the ubiquitination and degradation rate of RAD23B in GC cells (*P* < 0.05, Figures 4D and 4E). MG132 treatment significantly reversed the effect of PSMD7 silencing on RAD23B levels in AGS cells (*P* < 0.05, Supplementary Figure 7). Hence, PSMD7 stabilized RAD23B protein via deubiquitination in GC cells.

### RAD23B mediates the oncogenic role of PSMD7 in GC cells

**Figure 5:**
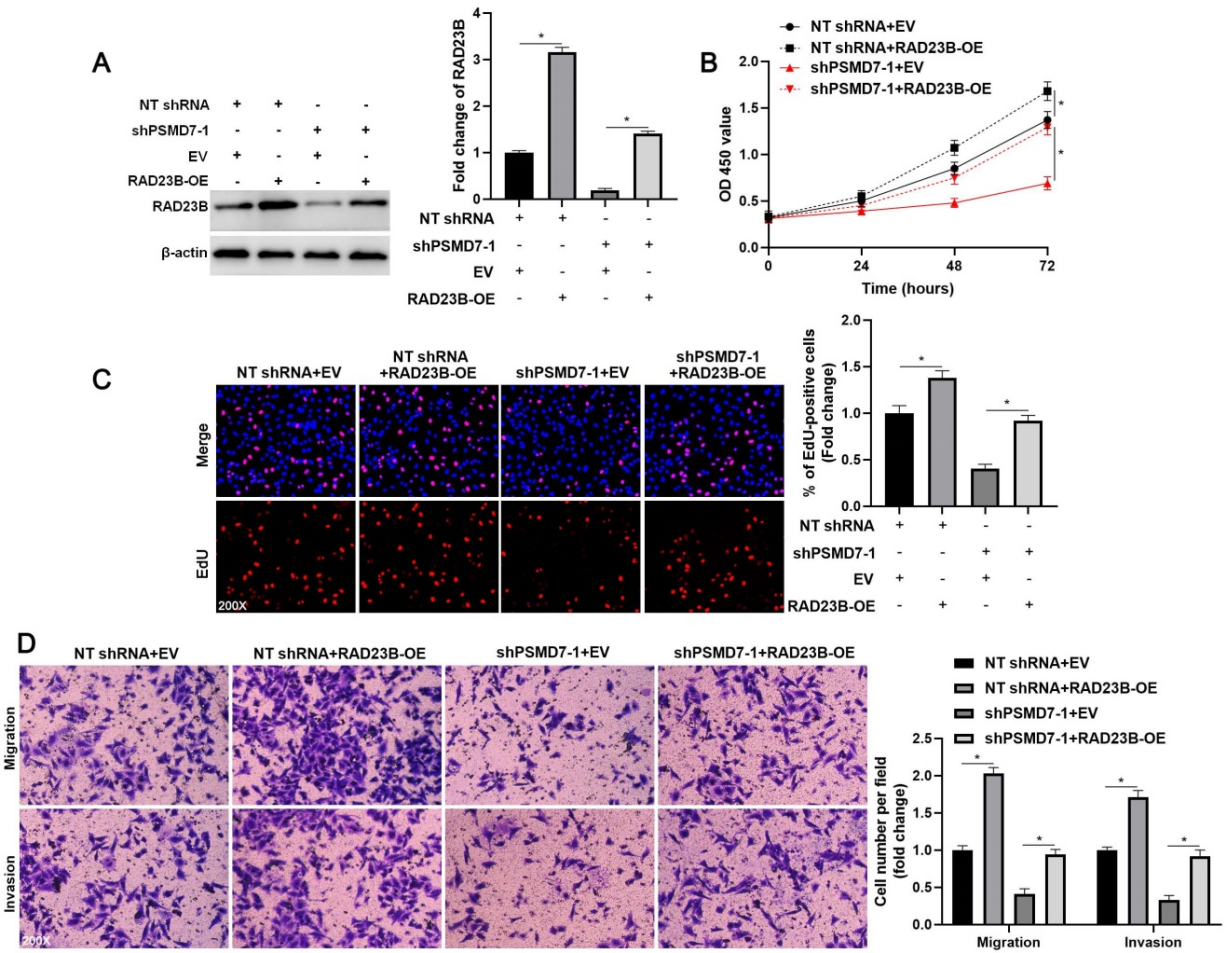
**RAD23B reverses the inhibitory role of PSMD7 knockdown in AGS cells**. (A) WB detection indicated that the RAD23B level was restored upon transfecting pcDNA3.1 vector carrying RAD23B cDNA into AGS cells. (B) MTT, (C) EdU, and (D) transwell assays indicated that RAD23B restoration abolished the suppressive effects of PSMD7 silencing on AGS cell proliferation, migration, and invasion. *P < 0.05.

To investigate the role of RAD23B in PSMD7-induced GC cell proliferation, migration, and invasion, we ectopically expressed RAD23B in AGS cells (*P* < 0.05, Figure 5A). RAD23B overexpression markedly enhanced the proliferative, migratory, and invasive abilities of AGS cells (*P* < 0.05, Figures 5B-5D). Furthermore, the inhibitory effect of PSMD7 knockdown on AGS cell proliferation was significantly reversed by RAD23B restoration (*P* < 0.05, Figures 5B and 5C). Moreover, RAD23B re-expression obviously increased the number of migrated and invaded cells in PSMD7-silenced AGS cells (*P* < 0.05, Figure 5D). Thus, RAD23B participated in PSMD7-enhanced malignant behaviors of GC cells.

### PSMD7 contributes to DDP resistance of GC cells *in vitro* and *in vivo*

**Figure 6:**
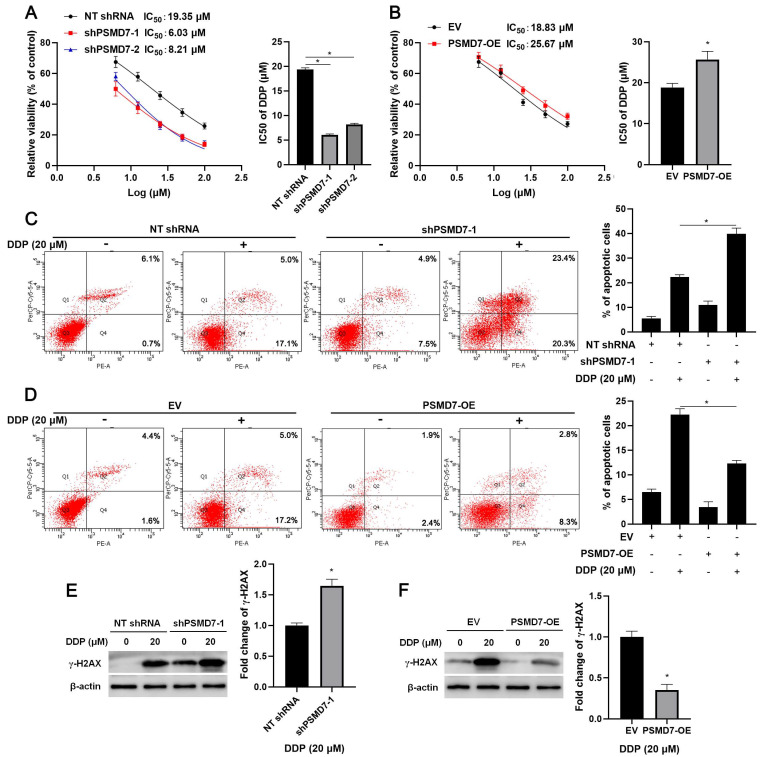
** PSMD7 maintained the resistance of GC cells to DDP**. (A) MTT assay demonstrated that PSMD7 knockdown increased the sensitivity of AGS cells to DDP. (B) PSMD7 overexpression reduced the sensitivity of AGS cells to DDP. (C) PSMD7 knockdown increased DDP-induced apoptosis of AGS cells. (D) PSMD7 overexpression decreased DDP-induced apoptosis of AGS cells. (E) PSMD7 knockdown increased DDP-induced γ-H2AX levels in AGS cells. (F) Ectopic expression of PSMD7 reduced DDP-induced γ-H2AX levels in AGS cells. *P < 0.05.

**Figure 7:**
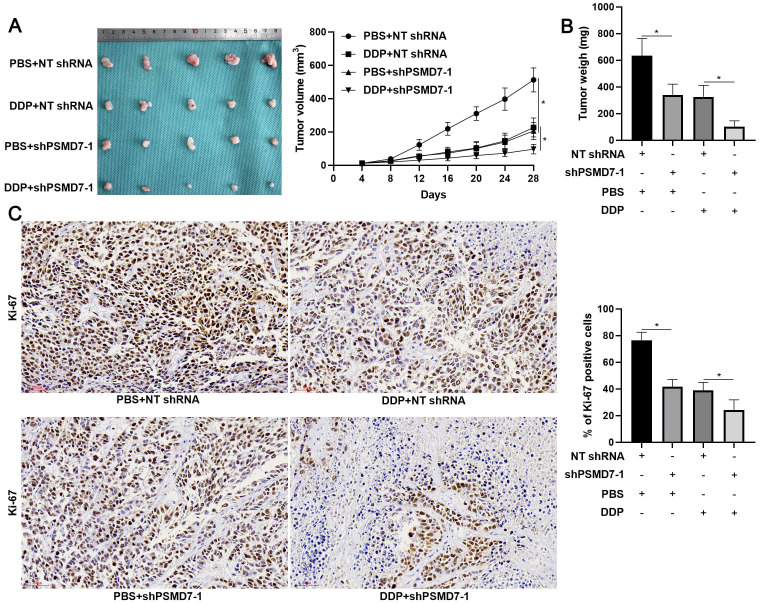
** PSMD7 knockdown enhances the efficiency of DDP treatment for tumor-bearing mice.** (A) AGS cells with or without PSMD7 knockdown were implanted into nude mice via subcutaneous injection. One week after implantation, the mice were treated with DDP (5 mg/kg) in PBS (thrice a week) via intraperitoneal injection. Tumor growth curves indicated that PSMD7 knockdown reduced tumor growth and enhanced DDP treatment efficiency for tumor-bearing mice. (B) The tumor weights in different treatment groups were measured. (C) PSMD7 knockdown significantly reduced the percentage of Ki-67-positive cells in xenograft tumor tissues. PSMD7 knockdown prominently enhanced the reduction in the number of Ki-67-positive cells upon DDP treatment. Scale bar: 50 μm. *P < 0.05.

Since RAD23B is implicated in chemotherapy drug-induced DNA damage, wherein it maintains XPC stability^25^, we aimed to study the role of PSMD7 in the chemoresistance of GC cells. After PSMD7 knockdown, the IC_50_ of DDP in AGS cells remarkably decreased (*P* < 0.05, Figure 6A). Conversely, the IC_50_ of DDP was significantly elevated by PSMD7 overexpression in AGS cells (*P* < 0.05, Figure 6B). Furthermore, PSMD7 knockdown enhanced, whereas PSMD7 overexpression weakened, DDP-induced apoptosis of AGS cells (*P* < 0.05, Figures 6C and 6D). Moreover, PSMD7 silencing upregulated, but the ectopic expression of PSMD7 downregulated, DDP-induced γ-H2AX protein levels in AGS cells (*P* < 0.05, Figures 6E and 6F). Next, the biological function of PSMD7 was confirmed *in vivo*. As shown in Figure 7A, PSMD7 knockdown significantly repressed GC tumor growth in nude mice (*P* < 0.05). Furthermore, PSMD7 silencing significantly enhanced the sensitivity of GC cells to DDP treatment *in vivo* (*P* < 0.05, Figures 7A and 7B). PSMD7 knockdown markedly reduced the percentage of Ki-67 positive cells but increased the number of TUNEL-stained cells in xenograft tumor tissues with or without DDP treatment (*P* < 0.05, Figure 7C and Supplementary Figure 8). Therefore, PSMD7 was involved in the DDP resistance of GC.

## Discussion

Aberrantly expressed DUBs are recognized as prognostic biomarkers and regulate the malignant behavior and therapeutic resistance of human cancers, including GC^10^, ^28^. The upregulation of PSMD7 and its association with poor prognosis in head and neck squamous cell carcinoma (HNSCC) and breast cancer have been reported^19^, ^20^, ^29^. In this study, we screened PSMD7 as an upregulated gene and prognostic biomarker in GC based on TCGA data. We further confirmed that PSMD7 protein levels were higher in GC tissues than in tumor-adjacent tissues. Positive expression of PSMD7 was associated with malignant clinical features and poor prognosis of GC. Hence, PSMD7 may be an indicator of the clinical outcomes of GC patients. A previous study showed that p65 transcriptionally activates COP9 signalosome 5 (CSN5) to mediate the deubiquitination and stability of programmed cell death-ligand 1 (PD-L1) in cancer cells^30^. Hypoxia-induced hypoxia-inducible factor-1α (HIF1α) is responsible for the transcription and expression of USP22, which maintains the stability and transcriptional activity of HIF1α via deubiquitination^31^. Therefore, the transcription factors involved in PSMD7 overexpression in GC require further investigation.

DUBs affect various malignant behaviors of cancer cells, including proliferation, invasion, angiogenesis, stemness, and chemoresistance, by deubiquitinating and stabilizing substrates^8^-^10^. Studies have demonstrated that PSMD7 silencing induces proliferation inhibition and apoptosis of ESCC cells, and results in cell cycle arrest, cell senescence, and apoptosis in breast cancer cells^18^, ^19^. As expected, we identified *PSMD7* as an oncogene in GC. PSMD7 knockdown repressed, whereas PSMD7 overexpression enhanced, the proliferation, migration, and invasion of GC cells. Furthermore, PSMD7 silencing inhibited GC tumor growth *in vivo*. A previous study indicated that PSMD7 knockdown increased the stability of p21 and p27 in breast cancer cells^19^. In this study, the PIPs and STRING databases were used to predict the interaction between PSMD7 and RAD23B. PSMD7 positively regulated the abundance of RAD23B and XPC protein in GC cells. Co-IP assays confirmed that PSMD7 interacted with RAD23B. PSMD7 knockdown increased the ubiquitination and degradation of RAD23B in GC cells. These data suggest that RAD23B is a novel substrate of PSMD7. Previous studies have demonstrated that RAD23B contributes to DNA damage repair via XPC^32^, ^33^. The role of RAD23B in different types of cancers is inconsistent. In breast cancer, RAD23B knockdown increases cell invasion and adhesion^34^. RAD23B reduces the sensitivity of prostate cancer cells to docetaxel^35^. RAD23B is expressed in circulating tumor cells (CTCs) from locally advanced rectal cancer patients who do not respond to neoadjuvant chemoradiation^36^. Notably, RAD23B was previously reported to confer radioresistance and chemoresistance onto GC cells^25^, ^26^. We found that the knockdown of PSMD7, an upstream regulator of RAD23B, enhanced, whereas its overexpression reduced, the sensitivity of GC cells to DDP. PSMD7 significantly inhibited DDP-induced apoptosis and γ-H2AX protein expression in GC cells. Moreover, PSMD7 knockdown suppressed the *in vivo* growth of GC cells and enhanced the efficiency of DDP treatment for tumor-bearing mice. Thus, PSMD7 promoted the malignant behavior and DDP resistance of GC cells by deubiquitinating and stabilizing RAD23B.

In summary, we firstly investigated the overexpression of PSMD7 in GC and verified that PSMD7 has the potential to predict poor prognosis. PSMD7 promoted cell proliferation, migration, and invasion, and enhanced GC cell resistance to DDP by deubiquitinating and stabilizing RAD23B. This study provided new insights into the mechanisms involved in the malignant progression and chemoresistance of GC. Targeting PSMD7 may improve the treatment and clinical outcomes of GC patients.

## Supplementary Material

Supplementary figures and table.

## Figures and Tables

**Table 1:** Correlations between PSMD7 expression and clinicopathologic characteristics of GC patients

Characteristics		n	PSMD7 expression		*P*
			Negative (n=33)	Positive (n=47)	
Age (years)	<60	39	17	22	0.678
	≥60	41	16	25	
Gender	Male	53	21	32	0.679
	Female	27	12	15	
Pathological differentiation	Well/moderate	36	20	16	0.019^*^
	Poor	44	13	31	
Depth of invasion	T1/T2	11	7	4	0.104
	T3/T4	69	26	43	
Lymph node metastasis	N0	20	11	9	0.149
	N1/N2/N3	60	22	38	
Distant metastasis	M0	76	32	44	0.639
	M1	4	1	3	
Tumor size (cm)	<5	35	20	15	0.011^*^
	≥5	45	13	32	
TNM stage	I+II	33	18	15	0.043^*^
	III+IV	47	15	32	

TNM, tumor-node-metastasis. ^*^Statistically significant.

## Abbreviations

GC: gastric cancer
PSMD7: proteasome 26S subunit, non-ATPase 7
DDP: cisplatin
DUBs: deubiquitinases
USP29: ubiquitin-specific protease 29
TGF-β1: transforming growth factor beta 1
hnRNPA1: heterogeneous nuclear ribonucleoprotein A1
CAFs: cancer-associated fibroblasts
JAMMs: JAMM/MPN domain-associated metallopeptidases
ESCC: esophageal squamous cell carcinoma
HNSCC: head and neck squamous cell carcinoma
CSN5: COP9 signalosome 5
PD-L1: programmed cell death-ligand 1
HIF-1α: hypoxia inducible factor-1α
CTCs: circulating tumor cells
