# Supplementary material for: Deubiquitinase PSMD7 promotes the proliferation, invasion, and cisplatin resistance of gastric cancer cells by stabilizing RAD23B
Source: Int J Biol Sci. 2021 Jul 25;17(13):3331–42. doi: 10.7150/ijbs.61128 (PMC8416741; doi:10.7150/ijbs.61128)
Supplement: Supplementary file 1 — Supplementary figures and table. [file ijbsv17p3331s1.pdf]

Supplementary Table 1. The primer sequences used in this study

Gene	Primer	Sequence (5′-3′)
PSMD7	forward	TTGGAGCAGAGGAAGCTGAG
	reverse	CTGACATCTGGCAGCAGGTT
RAD23B	forward	CTTCCTCCACCACCACAACT
	reverse	GGTGTCTCTGCTGGCTTTTC
β-actin	forward	CATGTACGTTGCTATCCAGGC
	reverse	CTCCTTAATGTCACGCACGAT

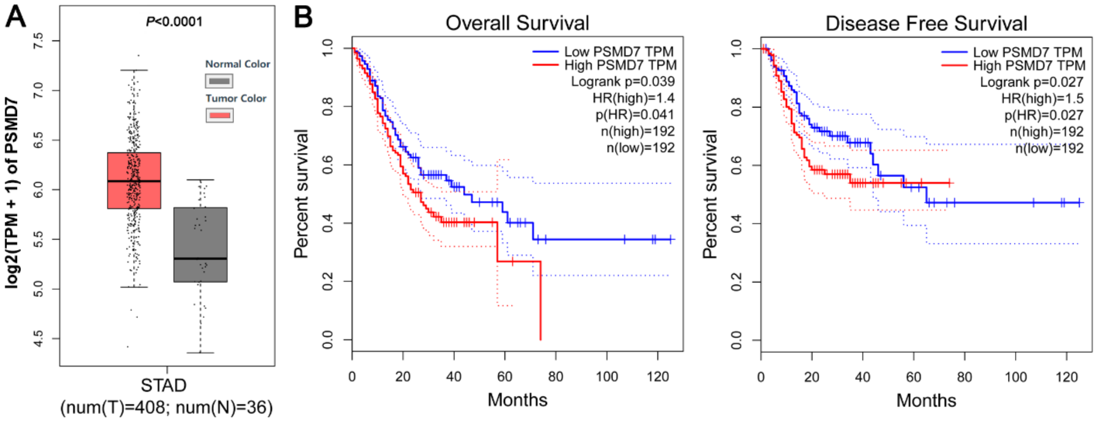

Supplementary Figure 1 The expression and prognostic significance of PSMD7 mRNA in GC. (A) TCGA-STAD data analysis using GEPIA web server revealed that the expression of PSMD7 mRNA in GC tissues was significantly higher than that in normal gastric tissues. (B) TCGA-STAD data analysis using GEPIA web server indicated that the high PSMD7 mRNA level predicted poor prognosis of GC.

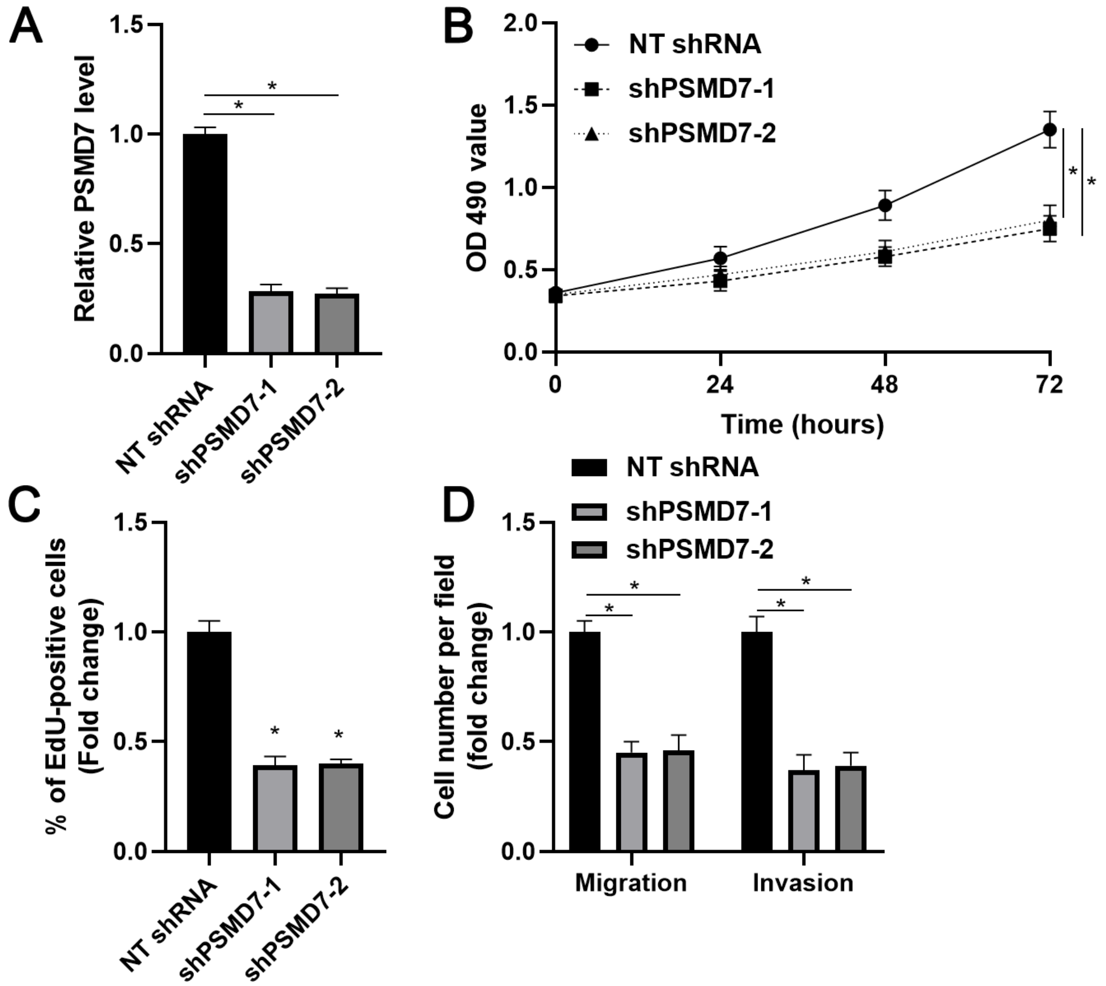

Supplementary Figure 2 PSMD7 knockdown represses the proliferation, migration, and invasion of SGC-7901 cells. (A) The level of PSMD7 in SGC-7901 cells transfected with PSMD7 shRNAs (shPSMD7-1 and shPSMD7-2) was significantly lower than that in cells transfected with non-targeting (NT) shRNA. (B) MTT, (C) EdU, and (D) transwell assays verified that PSMD7 knockdown inhibited the proliferation, migration, and invasion of SGC-7901 cells. *P<0.05.

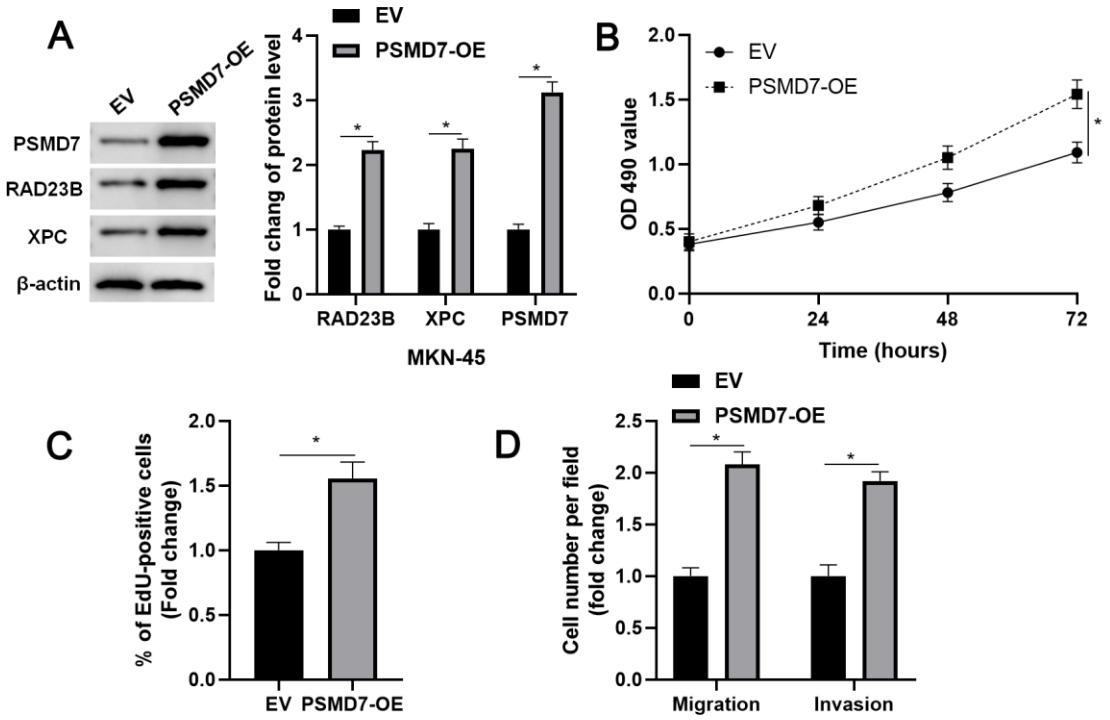

Supplementary Figure 3 PSMD7 overexpression enhanced the proliferation, migration, and invasion of MKN-45 cells. (A) The levels of PSMD7, RAD23B, and XPC in MKN-45 cells transfected with PSMD7-OE was significantly higher than those in cells transfected with EV. (B) MTT, (C) EdU, and (D) transwell assays verified that PSMD7 overexpression enhanced the proliferation, migration, and invasion of MKN-45 cells. **P*<0.05.

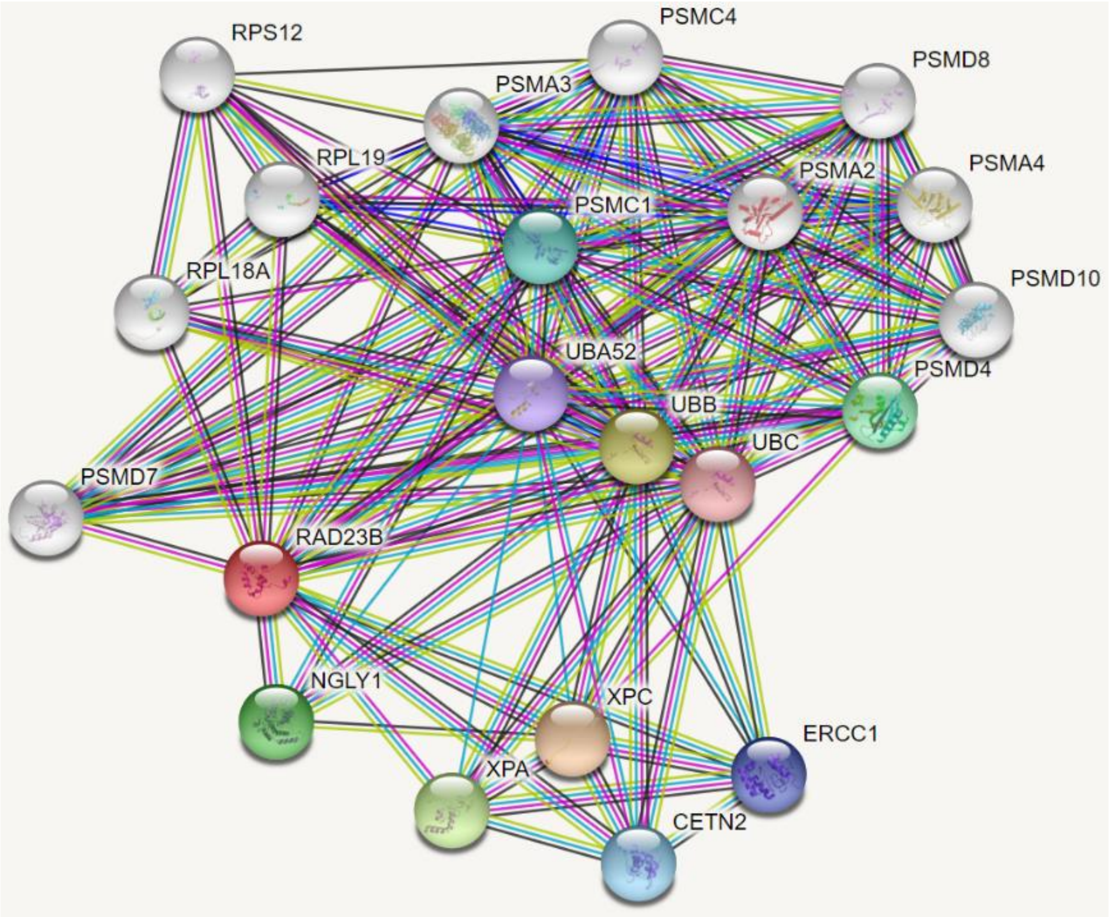

Supplementary Figure 4 STRING web server predicts the potential proteins interacted with PSMD7

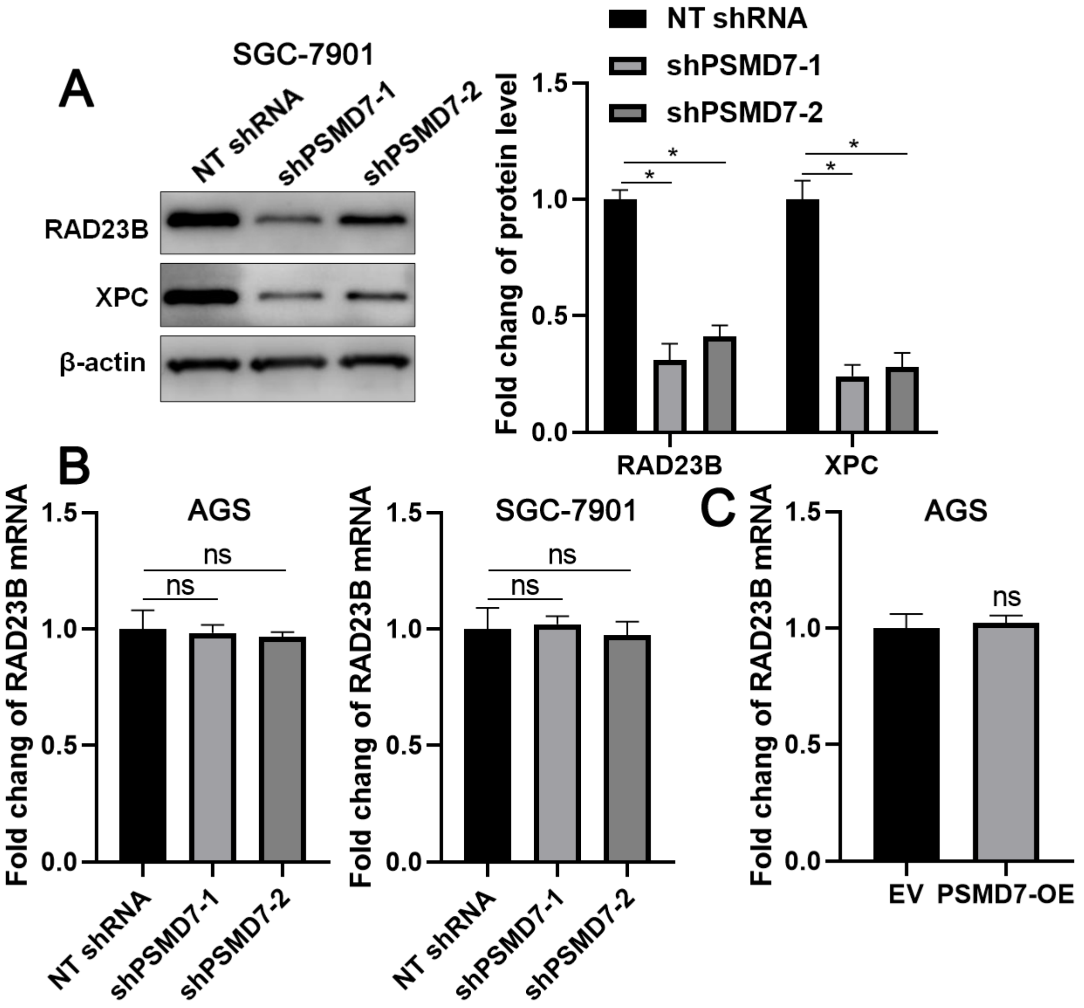

Supplementary Figure 5 PSMD7 regulates RAD23B protein rather than RAD23B mRNA in GC cells. (A) PSMD7 knockdown reduced the levels of RAD23B and XPC protein in SGC-7901 cells. (B) PSMD7 knockdown did not impact the RAD23B mRNA expression in AGS and SGC-7901 cells. (C) PSMD7 overexpression did not affect the RAD23B mRNA level in AGS cells. *P<0.05.

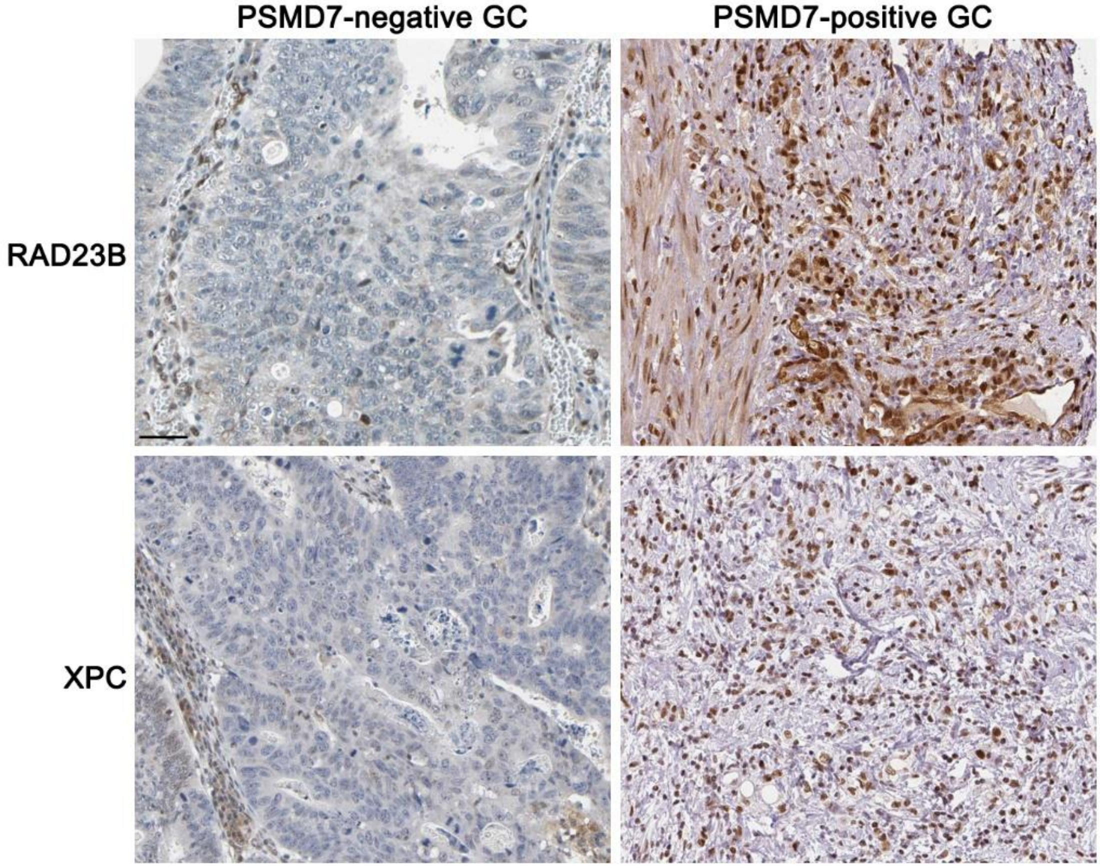

	PSMD7 (-)	PSMD7 (+)	*P*-value
RAD23B (-)	21	18	0.0256
RAD23B (+)	12	29	
XPC (-)	19	16	0.0367
XPC (+)	14	31	

Supplementary Figure 6 Immunostaining of RAD23B and XPC in GC tissues.

Scale bar: 50μm.

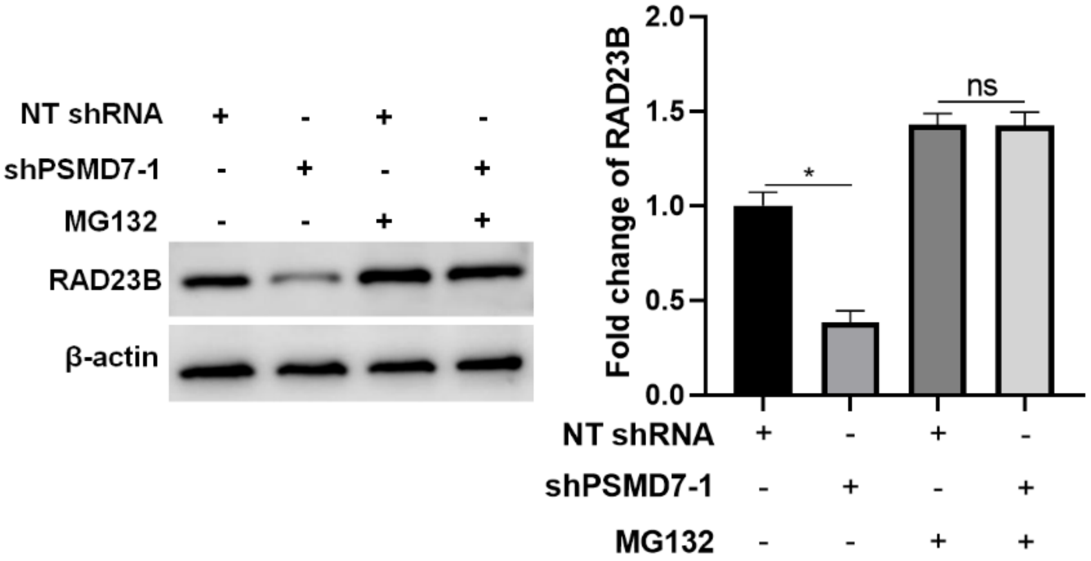

Supplementary Figure 7 MG132 treatment abolishes PSMD7 silencing-induced RAD23B downregulation in AGS cells. *P<0.05.

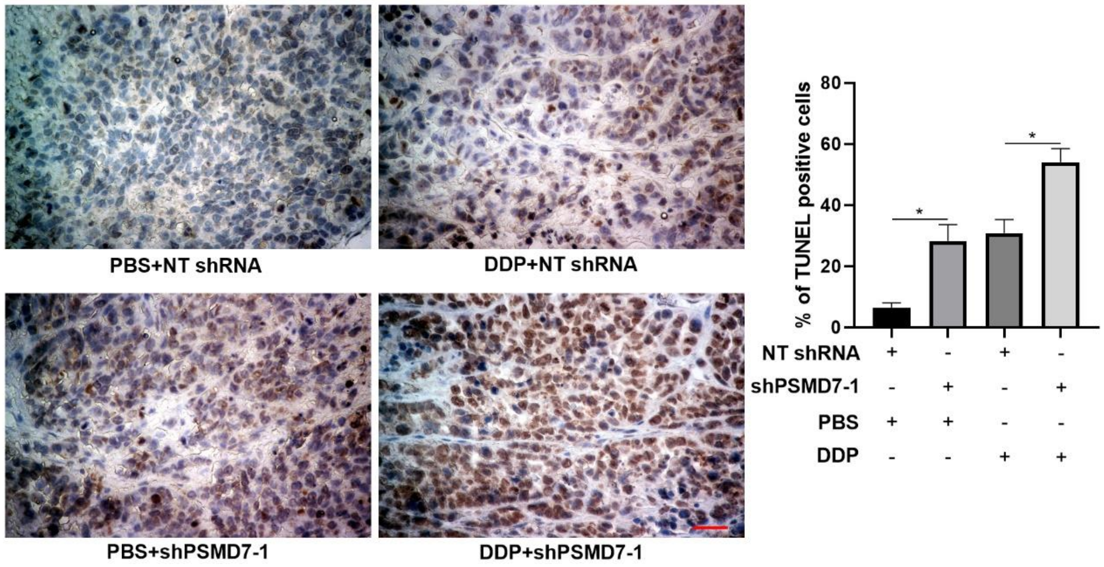

Supplementary Figure 8 PSMD7 knockdown enhances DDP-induced apoptosis in subcutaneous tumor tissues. Scale bar: 50μm. *P<0.05.
